# Effectiveness of Extracorporeal Shock Wave Therapy after Botulinum Toxin Injection for Post-Stroke Upper Extremity Spasticity: A Randomized Controlled Study

**DOI:** 10.3390/toxins16040197

**Published:** 2024-04-19

**Authors:** Junhee Lee, Seung Nam Yang

**Affiliations:** 1 Department of Physical Medicine and Rehabilitation, Ewha Womans University Mokdong Hospital, 1071, Anyangcheon-ro, Yangcheon-gu, Seoul 07985, Republic of Korea; jhl3728@gmail.com; 2 Department of Physical Medicine and Rehabilitation, Korea University College of Medicine, 73, Goryeodae-ro, Seongbuk-gu, Seoul 02841, Republic of Korea

**Keywords:** stroke, muscle spasticity, botulinum toxins, extracorporeal shock wave therapy

## Abstract

Post-stroke spasticity is a common complication that limits the functional performance of patients. Botulinum toxin (BTx) is an effective treatment for spasticity. Numerous researchers have applied extracorporeal shock wave therapy (ESWT) to address post-stroke spasticity, yielding positive clinical outcomes. We aimed to clarify the add-on effects of ESWT on BTx therapy for spasticity in patients with post-stroke. Sixteen eligible patients with upper extremity spasticity after stroke were recruited for this study. They were randomized to either a BTx with focused ESWT treatment group or a BTx alone group. Spasticity, measured using the modified Ashworth score (MAS) and modified Tardieu scale (MTS), showed statistically significant improvements in the elbow and wrist flexor muscles in both BTx + ESWT group and BTx alone groups. However, no significant differences were observed between the two groups with time flow. The BTx + ESWT group showed significantly decreased MAS of the finger flexors at follow-up and increased R1 (MTS) of the finger flexors at 3 weeks after treatment, which was not observed in the BTx alone group. This is the first study to identify the add-on effect of ESWT on BTx injections to improve post-stroke upper limb spasticity.

## 1. Introduction

Spasticity is a motor disorder characterized by a velocity-dependent increase in tonic stretch reflexes associated with an increased muscle tone and exaggerated tendon jerks [1]. Post-stroke spasticity is a common complication with a prevalence of 24.5% at 6 d, 26.7% at 6 weeks, and approximately 38% in the first year after stroke [2,3]. Although spasticity and muscle contracture are distinct problems, spasticity plays a role in the formation of contracture due to abnormal shortening of the soft tissue structure, restricting joint mobility and resulting in discomfort and rigidity [4,5]. Early management of post-stroke spasticity is important to avoid long-term complications, such as pain, pressure sores, muscle weakness, and joint contracture, which may lead to limitations in patients’ functional performance, activities of daily living, and community participation [6].

Current treatment options for post-stroke spasticity include physical therapy, exercise, oral spasticity medications, botulinum toxin (BTx) injections, and surgical management [7]. BTx is a protein produced by the bacterium *Clostridium botulinum* that acts on the peripheral neuromuscular junction by blocking acetylcholine release and altering muscle tone [8,9]. The effect of BTx is not permanent, and the frequency of injection and dosage are limited; therefore, various efforts have been made to sustain and increase its effects [10,11]. Repeated BTx injections are known to be performed at least 3 months apart [7,12].

Electrical shock wave therapy (ESWT) is defined as a series of single sonic pulses distinguished by high peaks, rapid pressure increases, and short durations of rapid prolongation [13]. Recently, ESWT has been shown to have good clinical results in the management of spasticity. One systematic review suggested high-level evidence that adjunct therapies may improve outcomes following botulinum toxin injection, especially ESWT, which led to improvements in the modified Ashworth score (MAS), spasm frequency scale, and pain [14]. Another review also showed level 1 evidence that ESWT is better than electrical stimulation for post-injection outcomes [15]. ESWT effectively reduces muscle tone in individuals with spastic limbs after stroke and is also considered a safe treatment tool free from undesirable side effects [16]. A study suggested that ESWT is a noninferior treatment alternative to BTx for post-stroke upper limb spasticity [17]. From this perspective, ESWT could be an adjuvant option after BTx injection for the treatment of spasticity to increase its effect. To the best of our knowledge, no study has identified the add-on effect of ESWT to conventional BTx therapy for patients with post-stroke. We hypothesized that additional ESWT treatment after BTx injection might further improve upper extremity spasticity and functional capacity.

## 2. Results

**Figure 1:**
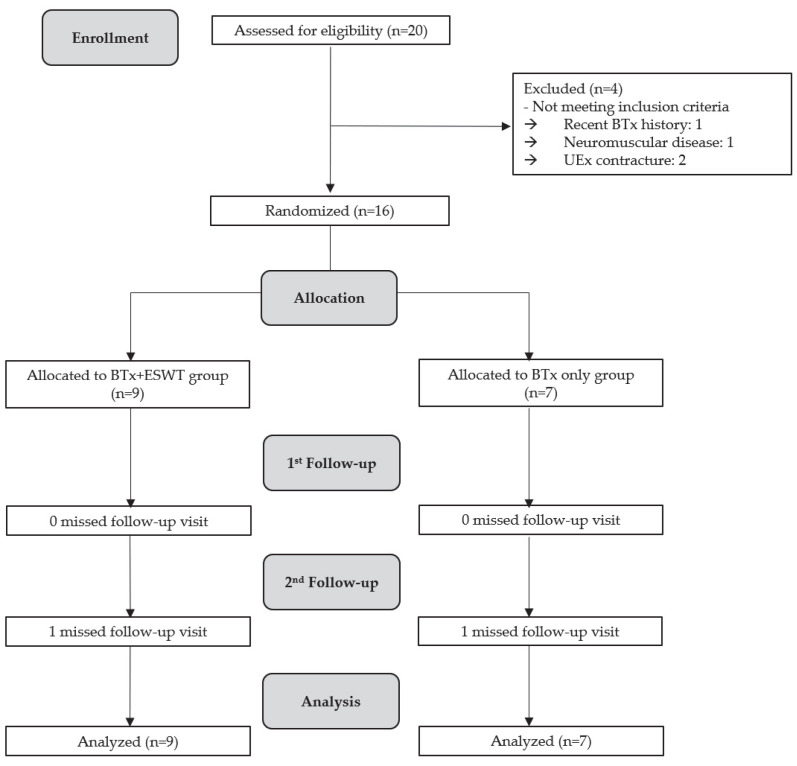
Flow diagram of patient recruitment in the study.

We consecutively enrolled 20 patients in this study. Four patients were excluded from the eligibility assessment. One patient received a recent BTx injection, two had contractures of their upper extremities, and one had a history of neuromuscular disease (Figure 1). Finally, 16 patients who were allocated in the study were randomly assigned to BTx + ESWT and BTx alone groups. Nine patients were assigned to the BTx + ESWT group, and seven were assigned to the BTx alone group. No significant difference was observed between the two groups in terms of baseline demographic characteristics and injected amount of BTx in each muscle, except for the time after stroke onset (Table 1). Six of the patients participating in the study were taking drugs for spasticity, such as dantrolene sodium and Baclofen. There was no significant difference in administration of drugs between the BTx with ESWT and the BTx alone groups. The average duration of rehabilitation for patients after the administration of botulinum toxin was 48.75 ± 82.61 min per week. There was no significant difference in treatment duration and frequency between the two groups. No serious adverse effects or complications occurred in the two groups during the study period.

### 2.1. Figures, Tables, and Schemes of Primary Outcomes

The MAS over time between the two groups showed a significant difference in time effect (*p* < 0.05) for the elbow, wrist, and finger flexors (Table 2) [18,19,20]. Otherwise, no significant group and time interaction was noted for the MAS measurements.

The MAS of both groups before treatment and during the follow-up period showed a statistically significant decrease at 3 weeks (*p* < 0.05) for the elbow and wrist flexors (Table 3). Additionally, a statistically significant decrease was noted in the MAS at 3 months (*p* < 0.05) in the wrist flexors. The BTx + ESWT group showed additional improvement in finger flexor spasticity at the follow-up time points (*p* < 0.05), which was not observed in the BTx alone group.

### 2.2. Secondary Outcomes

The modified Tardieu scale (MTS) over time between the two groups presented a significant difference with time effect (*p* < 0.05) in R1 of the elbow and wrist flexors and R2–R1 of the elbow and wrist flexors for both groups (Table 4) [21]. However, no significant group and time interaction were observed for the MTS measurements. 

The MTS scores in both groups at pretreatment and during follow-up showed statistically significant increases in R1 and decreases in R2–R1 were noted at 3 weeks (*p* < 0.05) in the elbow flexors and decreases in R2–R1 at 3 weeks (*p* < 0.05) in the wrist flexors (Table 5). A significant difference in R1 of the wrist and finger flexors between the two groups. The BTx + ESWT group showed an R1 increase in finger flexors at 3 weeks (*p* < 0.05), which was not observed in the BTx alone group.

No significant difference was noted in the time effect and group and time interaction for functional evaluations, such as the upper extremity Fugl-Meyer Assessment (UE-FMA) score, modified Barthel index (MBI) score, and Action Research Arm Test (ARAT) score, as reported (Table 6). In terms of UE-FMA, MBI, and ARAT, no statistically significant change was observed in functional evaluations in either group.

## 3. Discussion

Exercise is another important intervention for post-stroke spasticity [22,23]. Exercise can prevent the progression of muscle contractures and diminish hyperactivity of muscle tone. The purpose of stretching is to improve the viscoelastic properties of the muscle-tendon unit and increase extensibility. In addition, other anatomical structures can be put under tension, including tendons, or connective, vascular, dermal, and neural tissue [24]. Other physical therapies can be considered, such as the Bobath technique, which is based on the decrease of spasticity and promotive postural reflexes prior to facilitating voluntary activity in paretic muscles through attention to trunk posture as well as controlled muscle stretch at the limbs [7].

In addition to non-pharmacological management for spasticity after a stroke, a pharmacological approach with adjuvant therapies has been issued for several decades [14,25]. BTx is one of the most important and famous treatment choices for decreasing spasticity, and adjuvant therapies are expected to boost the effect of BTx injection [15]. 

The present study found that decreased spasticity was proven by the reduction of the MAS on finger flexors when ESWT was performed after BTx injection, but this was not observed in the BTx alone group. However, it was not found to have an additional effect on BTx injections into the elbow and wrist flexor muscles. Our study suggests that ESWT is an effective adjuvant treatment to increase the effectiveness of BTx treatment for post-stroke spasticity.

BTx injections are commonly used in the management of upper limb spasticity after a stroke and have been proven to be effective in alleviating spasticity and enhancing upper limb functionality [8]. The effects of BTx injections are temporary, and repeated injections are usually recommended to control continuous spasticity [12]. However, subsequent retreatments may produce fewer results. The development of neutralizing antibodies is commonly considered the primary factor contributing to treatment failure, as well as improper product handling, inappropriate dosing, and technique of injection [26]. Increases in neutralizing antibodies have been linked to larger doses per treatment, higher cumulative doses, and more frequent treatment schedules [27,28]. Several adjuvant treatments, along with the appropriate BTx technique, were used to achieve sufficient effects at lower doses of BTx [14].

Several adjuvant treatments have been suggested in combination with BTx to enhance effectiveness and reduce soft tissue contracture. Muscle stretching, adhesive taping, splinting/orthosis, and serial casting can be easily adjusted after BTx injection [15]. In a systematic review of related research, continuous posture by taping and casting led to better and longer-lasting effects on spasticity, gait function, and range of motion than stretching alone. The effectiveness of physical modalities as adjuvant treatments after BTx for spasticity management has been extensively documented in the literature. Various modalities, including ESWT, therapeutic ultrasound, vibration therapy, electrical stimulation, and transcutaneous electrical stimulation, have been studied [14,29,30,31,32,33,34,35]. One randomized trial focused on assessing the effectiveness of ESWT after BTx injections compared with electrical stimulation after BTx therapy for the management of focal upper limb spasticity in patients with stroke. Although electrical stimulation augments the diffusion of BTx, its effect with ESWT is boosted mechanically and topically by reducing muscle tone and inducing neovascularization in muscles. Due to these differences in the mechanism of action, this study concluded that ESWT enhanced the effect of BTx more than electrical stimulation by modulating the rheology of the muscle and neurotransmission at the neuromuscular junction [36]. 

There have been some explanations for the mechanisms by which ESWT improves spasticity. One suggestion is that ESWTs induce nitric oxide (NO) synthesis, which is critical for the production of new neuromuscular junctions in the peripheral nervous system and for various physiological functions of the central nervous system, including neurotransmission, memory, and synaptic plasticity. The synthesis of NO may lead to neovascularization, enhance tissue blood supply, and modulate interleukin secretion, thereby regulating inflammation and stimulating growth factors within a spastic muscle [29,37,38,39]. Another hypothesis is that ESWT may directly modulate the rheological properties of the spastic muscle. Mechanical shock or vibration from ESWT can disrupt the functional connection between actin and myosin, thereby decreasing the rigidity of connective tissues within a spastic muscle [29,37,38,40]. Additionally, ESWT has been reported to show antispastic effects by temporarily disturbing neuromuscular transmission by reducing acetylcholine receptors at neuromuscular junctions [41]. 

ESWT can be classified into two main modalities based on the wave propagation pattern: focused and radial shock wave therapies [29,42]. In focused shockwave therapy, waves are generated from the probe and converge at the target area. Conversely, radial shock wave devices concentrate their maximum energy at the probe tip and distribute it radially into the tissue. Recent studies have suggested that both focused and radial shock wave therapies are effective in reducing spasticity in stroke patients [43,44,45,46]. In our study, focused ESWT was used. Focused shock waves can penetrate deeper into tissues and focus their energy, whereas radial shock waves have the advantage of covering broader therapeutic areas. However, the clinical differences between radial and focused shock waves remain unclear. In a comparative study on the effects of focused and radial ESWT on spastic equinus in post-stroke patients, no difference was found between the two groups [29].

In the present study, we used 0.030 mJ/mm^2^ of an energy level with 4 Hz of frequency. Parameters of ESWT applied to spasticity in previous studies were heterogeneous. The energy levels varied between 0.03–0.30 mJ/mm^2^, and the frequencies ranged 4–8 Hz, with 4 Hz being the most commonly used frequency across the studies. The number of ESWT shots was more heterogeneous. We used 1000 shots, whereas other studies used a range of shots 1000–3000 [17,35,36,47,48]. Although more research is needed to fully understand the mechanisms of action and optimize treatment protocols, evidence from previous studies suggests that ESWT may offer benefits for individuals with post-stroke spasticity. However, it is essential for physicians to determine whether ESWT is appropriate, to determine the parameters of ESWT for a specific individual, and to discuss the potential risks and benefits.

A previous study compared the efficacy of ESWT and BTx in the treatment of post-stroke upper limb spasticity. Previous studies have investigated the effects of ESWT and BTx in the treatment of post-stroke upper limb spasticity [49,50]. In the present study, improvement in spasticity in the ESWT group was similar to that observed in the BTx injection group. The response rates did not differ significantly between the two groups. Additionally, a systematic review comparing the efficacy of BTx and ESWT in treating spasticity showed beneficial effects of both treatments. This review includes subjects with spasticity due to variable neurological diseases [17]. One systematic review compared the efficacies of ESWT and BTx in the management of spasticity [49]. Another systematic review and network meta-analysis clarified that both BTx injections and ESWT were effective in reducing post-stroke spasticity up to mid-term. The effectiveness of ESWT was comparable with that of BTx injections, with radial ESWT showing potential as the most effective treatment for reducing spasticity among BTx, focused ESWT, and radial ESWT [50].

Our study is valuable, as it is the first to demonstrate the effect of ESWT as an adjuvant treatment after BTx injection; however, it has some potential limitations. First, our study population was relatively small; therefore, this can act as a bias that reduces the reliability of the study. Second, our study design lacked a sham or noninterventional control group. Therefore, the beneficial effects of either the BTx + ESWT or the BTx alone group simply due to natural recovery cannot be ruled out. However, the extent of the improvement suggests that spontaneous recovery is unlikely. In addition, the effect of BTx on post-stroke upper limb spasticity is well known. Third, the intensity and duration of the ESWT were based on those reported in previous studies. It remains unclear whether a greater number of sessions or higher treatment intensity would have resulted in greater changes in outcomes or revealed greater or lesser differences between the two groups. Fourth, although the outcome evaluator did not know which group the participant belonged to, the patient was not blinded; therefore, it cannot be concluded that this had absolutely no effect. Fifth, the patients were followed up until 3 months since the duration of the effect of BTx is known to generally last for 3 months, but the results after that were not evaluated. Therefore, the subsequent effects could not be evaluated after 3 months. Finally, we used the MAS and MTS as outcome measures. Although these are commonly used tools for evaluating spasticity, they may not be sufficiently sensitive for detecting small differences. Considering these limitations, we expect that the following large, randomized, sham-controlled trials will compare the add-on effects of ESWT treatment on BTx injection therapy using precise outcome measures.

## 4. Conclusions

This is the first study to identify the add-on effect of focused ESWT on BTx injection to improve post-stroke upper extremity spasticity and functional capacity. BTx injection with adjuvant ESWT therapy showed distinguished improvement in spasticity of the distal flexor muscle of the upper extremity. Our study suggests future perspectives on the usefulness of ESWT as an effective adjuvant treatment to increase the effectiveness of BTx treatment for post-stroke patients with spasticity in clinical fields. This study was limited due to small group sizes; therefore, a larger scope of study is needed in the future to determine the additional effects of focused ESWT treatment on BTx injections.

## 5. Materials and Methods

This was a prospective, randomized, controlled study. Post-stroke patients with upper extremity spasticity were recruited from the Department of Physical Medicine and Rehabilitation, Korea University Guro Hospital, between August 2020 and June 2021. Inclusion criteria were as follows: (1) age >18 years and <80 years; (2) at least six weeks after stroke diagnosis; and (3) upper extremity (elbow, wrist, and finger flexors) spasticity MAS score >2. Exclusion criteria were as follows: (1) improper indication for BTx injection, such as myasthenia gravis, Eaton–Lambert syndrome, amyotrophic lateral sclerosis, and motor neuropathy; (2) previous contracture and/or deformity of the upper extremities; (3) concurrent peripheral neuropathy and/or myopathy; (4) recent changes in medication that are expected to affect the degree of spasticity; and (5) difficulty in participating in the study due to cognitive impairment. The study was approved by the institutional review board of the Korea University Guro Hospital (Protocol number: 2019GR0159, approval date: 8 May 2019), and was conducted in accordance with the Declaration of Helsinki. To increase the quality of reporting of this study, CONSORT guidelines were evaluated. Also, the study protocol was registered at ClinicalTrials.gov (NCT05889026).

After baseline demographic and clinical evaluations, eligible patients were randomly allocated to either the BTx injection with ESWT treatment (BTx + ESWT) group or the BTx injection alone group. Random assignment was performed using a random number table, and this work was conducted by an individual not involved in the patient recruitment.

All treatments were conducted by the same experienced physiatrist with 20 years of clinical experience in stroke-related spasticity, who was not involved in the baseline evaluation and further follow-up assessment. The proper arm muscles for BTx injection were selected after clinical assessment, and the location of the intramuscular injection was defined using electrostimulation guidance. BTx (Nabota^®^, Daewoong Pharmaceutical Co. Ltd., Seoul, Republic of Korea) in 0.9% sodium chloride solution was used for this study. The biceps brachii (BB), brachioradialis, pronator teres, flexor carpi radialis (FCR), flexor carpi ulnaris, flexor digitorum profundus, and flexor digitorum superficialis muscles were selected after individual evaluation. For the BTx + ESWT group, focused ESWT provided by Dornier Aries^®^ (Dornier MedTech, Wessling, Germany) were used. Additionally, ESWT was administered directly to the middle of the muscle bellies of the BB or FCR with 1000 shots (4 Hx, energy flux density 0.030 mJ/mm^2^) after BTx injection, once a day for 5 d. The participants were requested to continue their previous schedule of medication and rehabilitation programs.

Clinical assessments were conducted before treatment, and at 3 weeks and 3 months after the BTx injection. All three assessments were performed by an experienced physiatrist who was blinded to the treatment assignment. Adverse events were monitored throughout the study period.

Primary outcome measure was MAS for upper extremity spasticity. The spasticity of the elbow, wrist, and finger flexors was evaluated before treatment and at 3 weeks and 3 months after treatment. For convenience, an MAS grade of 1+ was matched to two points, and grades 2, 3, and 4 were matched to three, four, and five points, respectively. Secondary outcome measures included improvement in the MTS score of the spastic upper extremity muscles, UE-FMA score, MBI score, and ARAT score. The UE-FMA was evaluated before and at 3 weeks after treatment. Other secondary outcome measures were assessed before treatment and at 3 weeks and 3 months after treatment.

### Statistical Analysis

Descriptive data are presented as medians with interquartile ranges for continuous and categorical variables. Baseline demographic and clinical variables were compared between the treatment groups using the Mann–Whitney U test for continuous data and chi-square test for categorical data.

The primary and secondary outcome measures were investigated using repeated-measures analysis of variance for the overall effect. These factors included group (BTx + ESWT vs. BTx alone) and time (preinjection, 3 weeks after injection, and 3 months after injection). Differences between the two groups over time were studied using the interaction term, group × time.

The Wilcoxon signed-rank test was performed to determine the differences in primary and secondary outcome measures between preinjection and at 3 weeks, and 3 months after injection. Statistical significance was considered at *p* < 0.05.

Statistical analyses were performed using SPSS version 28.0 software (SPSS Inc., Chicago, IL, USA). The required minimum sample size was calculated with a 5% significance level, 95% power, effect size (2.0), and two groups for the Mann–Whitney test using G*Power 3.1.9.2 software.

## Figures and Tables

**Table 1:** Baseline demographic characteristics.

Variables	BTx + ESWT Group N = 9	BTx Alone Group N = 7	*p*-Value
Age (years)	59 (37–62.5)	52 (42–62)	0.958
Sex			0.392
Male (%)	7 (77.8)	4 (57.1)	
Female (%)	2 (22.2)	3 (42.9)	
Height (cm)	170 (166–174)	173 (161–176)	0.874
Weight (kg)	72 (62–80)	75 (65–87)	0.523
Time after stroke onset (month)	39 (28–106.5)	18 (5–25)	0.015 *
Stroke subtype			0.356
Hemorrhagic (%)	5 (55.6)	3 (42.9)	
Ischemic (%)	4 (44.4)	4 (57.1)	
Affected side			0.705
Right (%)	3 (33.3)	3 (42.9)	
Left (%)	6 (66.7)	4 (57.1)	
Amount of injected BTxA (unit)			
BB	55.0 (42.5–67.5)	50.0 (35.0–57.5)	0.432
BR	50.0 (40.0–65.0)	50.0 (40.0–50.0)	0.344
PT	40.0 (350–50.0)	40.0 (30.0–70.0)	0.761
FCR	50.0 (50.0–50.0)	45.0 (40.0–50.0)	0.261
FCU	30.0 (25.0–35.0)	60.0 (30.0–40.0)	0.491
FDP	50.0 (50.0–50.0)	50.0 (50.0–50.0)	1.000
FDS	30.0 (30.0–45.0)	40.0 (30.0–50.0)	0.576

Continuous data are presented as medians with interquartile ranges, and categorical data as numbers (%). Abbreviations: BTx, botulinum toxin; BB, biceps brachii; BR, brachioradialis; PT, pronator teres; FCR, flexor carpi radialis; FCU, flexor carpi ulnaris; FDP, flexor digitorum profundus; FDS, flexor digitorum superficialis. * Significant at *p* < 0.05, using the Mann–Whitney U test or chi-square test.

**Table 2:** Primary outcomes: MAS along time between BTx + ESWT and BTx alone groups.

	BTx + ESWT Group			BTx Alone Group			*p*-Value	
	Pre Injection	3 Weeks after Injection	3 Months after Injection	Pre Injection	3 Weeks after Injection	3 Months after Injection	P1 (Time)	P2 (Time × Group)
Elbow flexors	2 (1.5–3)	1 (0–1)	1 (1–2)	2 (1–2)	1 (1–1)	1 (0.75–1.25)	<0.001 *	0.207
Wrist flexors	3 (2.5–3)	1 (0.5–1.5)	1 (0–1.75)	3 (3–4)	1 (1–1)	1 (0.75–1.5)	<0.001 *	0.622
Finger flexors	3 (1–3.5)	1 (0.5–1)	1 (0–2)	3 (0.5–3.5)	1 (0.5–1.5)	1 (0.25–1)	<0.001 *	1.000

Values are presented as medians (interquartile ranges). Abbreviations: MAS, modified Ashworth scale; BTx, botulinum toxin; ESWT, extracorporeal shock wave therapy. P1: *p*-value of time effect, P2: *p*-value of time × group interaction. * Significant at *p* < 0.05, using repeated measures analysis of variance.

**Table 3:** Primary outcomes at different time points.

	Elbow Flexors		Wrist Flexors		Finger Flexors	
	MAS	*p*-Value	MAS	*p*-Value	MAS	*p*-Value
BTx + ESWT group						
Preinjection	2 (1.5–3)	-	3 (2.5–3)	-	3 (1–3.5)	-
3 weeks after injection	1 (0–1)	0.006 *	1 (0.5–1.5)	0.014 *	1 (0.5–1)	0.041 *
3 months after injection	1 (1–2)	0.063	1 (0–1.75)	0.026 *	1 (0–2)	0.026 *
BTx alone group						
Preinjection	2 (1–2)	-	3 (3–4)	-	3 (0.5–3.5)	-
3 weeks after injection	1 (1–1)	0.034 *	1 (1–1)	0.016 *	1 (0.5–1.5)	0.141
3 months after injection	1 (0.75–1.25)	0.083	1 (0.75–1.5)	0.038 *	1 (0.25–1)	0.102

Abbreviations: MAS, modified Ashworth scale; BTx, botulinum toxin; ESWT, extracorporeal shock wave therapy. Values of MAS are medians (interquartile range). *p*-values at 3 weeks and 3 months were calculated and compared with preinjection values in both groups. * Significant at *p* < 0.05, using Wilcoxon signed-rank test.

**Table 4:** Secondary outcomes: MTS along time between BTx + ESWT and BTx alone groups.

			BTx + ESWT Group			BTx Alone Group			*p*-Value	
			Preinjection	3 Weeks after Injection	3 Months after Injection	Preinjection	3 Weeks after Injection	3 Months after Injection	P1 (Time)	P2 (Time × Group)
MTS	R1	Elbow flexors	95.00 (67.50–117.50)	130.00 (100.00–140.00)	82.50 (60.00–107.50)	110.00 (60.00–130.00)	120.00 (100.00–150.00)	110.00 (73.75–130.00)	0.001 *	0.270
		Wrist flexors	95.00 (87.50–107.50)	120.00 (95.00–130.00)	100.00 (85.00–117.50)	90.00 (85.00–110.00)	140.00 (100.00–150.00)	115.00 (106.25–127.50)	0.017 *	0.513
		Finger flexors	40.00 (20.00–45.00)	55.00 (30.00–70.00)	40.00 (30.00–80.00)	50.00 (−3.75–88.75)	50.00 (20.00–90.00)	90.00 (80.00–90.00)	0.158	0.183
	R2–R1	Elbow flexors	30.00 (22.50–62.50)	0 (0–32.50)	42.50 (20.00–68.75)	40.00 (20.00–80.00)	20.00 (0–40.00)	37.50 (15.00–65.00)	<0.001 *	0.554
		Wrist flexors	25.00 (20.00–42.50)	5.00 (0–20.00)	20.00 (3.75–33.75)	25.00 (10.00–45.00)	10.00 (0–20.00)	20.00 (11.25–30.00)	0.001 *	0.908
		Finger flexors	20.00 (10.00–40.00)	0 (0–10.00)	10.00 (0–25.00)	7.50 (1.25–25.00)	10.00 (0–10.00)	10.00 (0–20.00)	0.205	0.650

Values are presented as medians (interquartile ranges). Abbreviations: MTS, modified Tardieu scale; BTx, botulinum toxin; ESWT, extracorporeal shock wave therapy. P1: *p*-value of time effect, P2: *p*-value of time × group interaction. * Significant at *p* < 0.05, using repeated measures analysis of variance.

**Table 5:** Secondary outcomes: MTS at different time points.

	Elbow Flexors				Wrist Flexors				Finger Flexors			
	R1	*p*-Value	R2-R1	*p*-Value	R1	*p*-Value	R2-R1	*p*-Value	R1	*p*-Value	R2-–R1	*p*-Value
BTx + ESWT												
Preinjection	95.00 (67.5–117.5)	-	30.00 (22.50–62.50)	-	95.00 (87.50–107.50)	-	25.00 (20.00–42.50)	-	40.00 (20.00–45.00)	-	20.00 (10.00–40.00)	-
3 weeks after injection	130.00 (100.0–140.0)	0.007 *	0 (0–32.50)	0.007 *	120.00 (95.00–130.00)	0.011 *	5.00 (0–20.00)	0.015 *	55.00 (30.00–70.00)	0.018 *	0 (0–10.00)	0.058
3 months after injection	82.50 (60.0–107.5)	0.933	42.50 (20.00–68.75)	0.933	100.00 (85.00–117.50)	0.674	20.00 (3.75–33.75)	0.203	40.00 (30.00–80.00)	0.343	10.00 (0–25.00)	0.244
BTx alone												
Preinjection	110.0 (60.0–130.0)	-	40.00 (20.00–80.00)	-	90.00 (85.00–110.00)	-	25.00 (10.00–45.00)	-	50.00 (−3.75–88.75)	-	7.50 (1.25–25.00)	-
3 weeks After injection	120.0 (100.0–150.0)	0.043 *	20.00 (0–40.00)	0.043 *	140.00 (100.00–150.00)	0.603	10.00 (0–20.00)	0.042 *	50.00 (20.00–90.00)	0.655	10.00 (0–10.00)	0.317
3 months after injection	110.0 (73.8–130.00)	0.713	37.50 (15.00–65.00)	0.715	115.00 (106.25–127.50)	0.008	20.00 (11.25–30.00)	0.225	90.00 (80.00–90.00)	0.655	10.00 (0–20.00)	0.655

Values are presented as medians (interquartile ranges). Abbreviations: MTS, modified Tardieu scale; BTx, botulinum toxin; ESWT, extracorporeal shock wave therapy; IQR, inter-quartile range; t_0_, preinjection; t_1_, 3 weeks after injection; t_2_, 3 months after injection. *p*-values at 3 weeks and 3 months were calculated and compared with preinjection values in both groups. * Significant at *p* < 0.05, using Wilcoxon signed-rank test.

**Table 6:** Secondary outcomes: functional evaluations along time between BTx + ESWT and BTx alone groups.

		BTx + ESWT Group			BTx Alone Group			*p*-Value	
		Preinjection	3 Weeks after Injection	3 Months after Injection	Preinjection	3 Weeks after Injection	3 Months after Injection	P1 (Time)	P2 (Time × Group)
Functional evaluations	UE-FMA	13.0 (7.5–26.0)	.	18.0 (14.0–35.25)	11.0 (9.0–28.0)	.	14.0 (8.75–20.25)	0.916	0.332
	MBI	87.5 (85.25–88.0)	86.0 (81.0–88.0)	86.5 (83.5–88.0)	67.0 (62.0–77.0)	67.0 (62.0–77.0)	72.5 (55.25–80.75)	0.129	0.105
	ARAT	19.0 (3.0–20.0)	19.0 (3.0–25.0)	19.0 (5.75–28.25)	7.0 (3.0–20.0)	7.0 (3.0–20.0)	5.0 (2.75–19.25)	0.554	0.581

Values are presented as medians (interquartile ranges). Abbreviations: BTx, botulinum toxin; ESWT, extracorporeal shock wave therapy; UE, upper extremity; FMA, Fugl-Meyer assessment; MBI, modified Barthel index; ARAT, Action Research Arm Test. P1: *p*-value of time effect, P2: *p*-value of time × group interaction.

## Data Availability

The datasets generated and/or analyzed during the current study are not publicly available because they include the patients’ personal and sensitive information, but are available from the corresponding author upon reasonable request.
